# Syncrip/hnRNP Q influences synaptic transmission and regulates BMP signaling at the *Drosophila* neuromuscular synapse

**DOI:** 10.1242/bio.20149027

**Published:** 2014-08-29

**Authors:** James M. Halstead, Yong Qi Lin, Lita Durraine, Russell S. Hamilton, Graeme Ball, Greg G. Neely, Hugo J. Bellen, Ilan Davis

**Affiliations:** 1 Department of Biochemistry, South Parks Road, The University of Oxford, Oxford OX1 3QU, UK; 2 Friedrich Miescher Institute for Biomedical Research, Basel 4058, Switzerland; 3 Howard Hughes Medical Institute, Department of Molecular and Human Genetics, Department of Neuroscience, Program in Developmental Biology, Neurological Research Institute at Baylor College of Medicine, Houston, TX 77030, USA; 4 Neuroscience Program, Garvan Institute of Medical Research, Darlinghurst, Sydney, NSW 2010, Australia; 5 Micron Imaging Facility, Department of Biochemistry, South Parks Road, The University of Oxford, Oxford OX1 3QU, UK

**Keywords:** Syncrip, *Drosophila*, Localized translation, Synaptic transmission, mRNA localization neuromuscular junction

## Abstract

Synaptic plasticity involves the modulation of synaptic connections in response to neuronal activity via multiple pathways. One mechanism modulates synaptic transmission by retrograde signals from the post-synapse that influence the probability of vesicle release in the pre-synapse. Despite its importance, very few factors required for the expression of retrograde signals, and proper synaptic transmission, have been identified. Here, we identify the conserved RNA binding protein Syncrip as a new factor that modulates the efficiency of vesicle release from the motoneuron and is required for correct synapse structure. We show that *syncrip* is required genetically and its protein product is detected only in the muscle and not in the motoneuron itself. This unexpected non-autonomy is at least partly explained by the fact that Syncrip modulates retrograde BMP signals from the muscle back to the motoneuron. We show that Syncrip influences the levels of the Bone Morphogenic Protein ligand Glass Bottom Boat from the post-synapse and regulates the pre-synapse. Our results highlight the RNA-binding protein Syncrip as a novel regulator of synaptic output. Given its known role in regulating translation, we propose that Syncrip is important for maintaining a balance between the strength of presynaptic vesicle release and postsynaptic translation.

## INTRODUCTION

Neuronal communication depends on the release of neurotransmitters from the pre-synaptic terminal that causes rapid depolarization in the postsynaptic cell. In tandem, retrograde signals emanating from the post-synapse signal back to the pre-synapse. These signals modulate synaptic output through changes in the structure and function of the pre-synaptic terminals. The retrograde signals in flies are crucial for regulating local synaptic strength during development (Keshishian and Kim, 2004), homeostasis (Goold and Davis, 2007), and synaptic plasticity (Tao and Poo, 2001). In mammalian central neurons, the release of retrograde signals from the dendrites has been implicated in long term potentiation and synapse growth (Lledo et al., 1998), negative feedback (Magnusson et al., 2008), neuronal development (Fitzsimonds and Poo, 1998) and systemic signaling (Ludwig et al., 2002). However, despite their importance, relatively few factors are known to regulate the expression of retrograde signals, and little is known about how retrograde signaling is coordinated with other synaptic processes, such as local translation.

The *Drosophila* third instar neuromuscular junction (NMJ) is an excellent model to study synaptic function (Keshishian et al., 1996) and significant progress has been made in discovering some of the players involved in retrograde signaling. The rapid growth of the muscle during the larval stage requires a concomitant expansion of the neuromuscular synapse to maintain contraction efficacy. The ability to correlate changes on either side of the synapse with neuronal activity has revealed that retrograde signaling regulates plastic growth and homeostasis at the larval NMJ (Giagtzoglou et al., 2009; McCabe et al., 2003; McCabe et al., 2004; Paradis et al., 2001; Frank et al., 2006; Korkut et al., 2013). While the details of the retrograde signaling pathway has remained elusive, genetic and pharmacological studies have implicated calcium signaling (Neveu and Zucker, 1996; Frank et al., 2006), nuclear import pathways (Giagtzoglou et al., 2009), presynaptic exosome secretion (Korkut et al., 2013), postsynaptic vesicle trafficking via Synaptotagmin family members (Yoshihara et al., 2005), and Bone Morphogenic Protein growth factor secretion (Goold and Davis, 2007).

The Bone Morphogenic Protein (BMP) pathway contains members of the conserved transforming growth factor β (TGF-β) family and is one of the best characterized retrograde signaling pathways. The retrograde BMP ligand, Glass Bottom Boat (GBB) is secreted from the muscle and received by presynaptic receptors, which in turn phosphorylate the transcription factor Mothers Against Decapentaplegic (MAD) and interact with LIM kinase 1, which act in tandem to contribute to presynaptic stability and growth (reviewed by Zwijsen et al., 2003; Keshishian and Kim, 2004) (Eaton and Davis, 2005; Goold and Davis, 2007). P-MAD is then trafficked to the neuron nucleus where it programs transcription (Massagué and Wotton, 2000). BMP retrograde signaling serves to stabilise synapse structure and neurotransmission (Aberle et al., 2002; Marqués et al., 2002; McCabe et al., 2004; Berke et al., 2013). This conserved signaling pathway is central to the development and function of synapses, and misregulation is associated with a number of diseases (Bayat et al., 2011; Cai et al., 2012). Despite this, little is known about the factors that serve to regulate the BMP signaling pathway and coordinate it with other processes in the synapse.

Genetic studies suggest that retrograde signaling proceeds through a complex pathway involving multiple processes. However, one class of gene that has not been well examined in retrograde signaling are the RNA-binding proteins (RBPs). This is surprising, as RBPs are highly expressed in neuronal tissues and are known to be central to the long-term changes that facilitate synapse plasticity through localised translation (Wang et al., 2010). Moreover, recent genome-wide analysis has revealed that RBPs can participate in multiple cell processes auxiliary to mRNA metabolism (Castello et al., 2012).

We have identified a new factor in retrograde signaling, Syncrip (Syp), which is required in the muscle to influence vesicle release and membrane integrity pre-synaptically. Syp is the fly homolog of hnRNP Q/SYNaptotagmin-binding Cytoplasmic RNA-Interacting Protein (SYNCRIP) and is a highly conserved heterogenous nuclear ribonucleoprotein (hnRNP) (Mizutani et al., 2000; Bannai et al., 2004; McDermott et al., 2012). We identified Syp in a biochemical screen for proteins that associate with the *gurken* localization signal (Van De Bor et al., 2005), an RNA signal that is necessary and sufficient for the localization of *gurken* mRNA to determine axial polarity in the oocyte and future embryo (McDermott et al., 2012). We found that Syp was required for mRNA localization and local translation of *gurken* mRNA in oogenesis. Syp has also been detected in mammalian dendrites, where it is found in trafficked RNP granules containing mRNAs encoding synaptic receptors and non-coding regulatory RNAs (Duning et al., 2008; Bannai et al., 2004). Moreover, *in vitro*, mammalian SYNCRIP competes with Poly(A) binding proteins to inhibit translation (Svitkin et al., 2013) and is required to regulate dendritic morphology (Chen et al., 2012).

Here, we show that *Drosophila* Syp is present in the muscle and required for correct vesicle biogenesis, docking, and neurotransmitter release from the pre-synapse. This unexpected non-autonomous requirement for an RNA binding protein is explained by the fact that Syp is necessary for the correct levels of the canonical BMP signaling pathway in both muscle and neuron. We find that upregulation of BMP signaling in *syp* mutants correlates with aberrant synapse structure and function. Our results suggest that the conserved RNA binding protein Syp regulates synaptic output via retrograde signaling. Given that Syp is thought to have a known role in regulating translation, we propose that it serves to coordinate synaptic efficacy through retrograde signaling with postsynaptic localised translation.

## MATERIALS AND METHODS

### *Drosophila* strains and genetics

Stocks were raised on standard cornmeal agar medium at 25°C. Wildtype was Oregon R (OrR). *syncrip (syp)* null alleles were *syp^286^* (PBac{RB}CG17838e00286 insertion line) and *syp^Df124^* (*Df (3R)BSC124* (*Df(3R)BSC124*; Bloomington Deletion Project, Bloomington Stock Centre). It was not possible to express the 17 different isoforms transcribed from the Syp gene by GAL4 drivers. Expression of a single Syp isoform (F) in muscle did not recapitulate Syp distribution and perturbed neuromuscular structure (data not shown). Instead, a genomic rescue *syp^Rescue^* construct was generated with a fosmid (FlyFos024580; http://flybase.org/cgi-bin/gbrowse/dmel/?name = 3R:16603660..16635842) containing the endogenous Syp promoter and covering all Syp isoforms except A and H. The construct was inserted at the attP40 on chromosome 2 (insertion line – Bloomington 25709). *syp^Rescue^* was expressed in the *syp^286/Df124^* background.

### Electrophysiology and FM1-43 dye uptake experiments

Wandering third instar larvae were dissected in ice-cold 0.25 mM calcium HL-3 solution (containing 70 mM NaCl, 5 mM KCl, 20 mM MgCl_2_, 10 mM NaHCO_3_, 5 mM Trehalose, 5 mM HEPES, 115 mM Sucrose; pH 7.2). Dissected larvae, then, rinsed three times with HL-3 with 0.5 mM Ca^2+^, and then incubated for at least 3 min before recording. All intracellular recordings were made at muscle 6 of abdominal segment A3, by using with sharp glass electrodes filled with a 2:1 mixture of 2 M potassium acetate to 2 M potassium chloride (resistance of 32–40 MΩ). Both Excitatory Junction Potential (EJPs) and Miniature EJPs (mEJPs) were amplified with an Axonclamp 2B amplifier in bridge mode under the control of Clampex 8.2 (Axon Instruments Inc.). All experiments were performed at room temperature (20–22°C). EJPs were evoked by directly stimulating segmental nerve innervating either hemisegment A3 through a glass capillary electrode (internal diameter, ∼10 µm) at 0.2 Hz. The applied currents were 6 µA ± 3 with fixed stimulus duration at 0.3 ms which was 50% larger than that required to activate both 1b and 1s boutons on recording muscles. Twenty to thirty evoked EJPs were recorded and analyzed for each animal (n number refers to the number of animals tested). Miniature EJPs (mEJPs) events were collected for 5 minutes (n number refers to the number of animals tested). Data were collected only when resting membrane potential below −62 mV, however, those data were rejected if resting membrane potential were shifted more than ± 5 mV during the course of experiment. In addition, only one muscle per larvae was recorded in each individual experiment. For paired-pulses protocol, two evoked stimuli were delivered at a short inter-pulse interval of 50 ms (ΔT), repetitively five times with rate of 0.008 Hz (every 2 minutes). EJPs and paired-pulse stimulation were analyzed with Clampfit 9.2 software (Axon Instruments). Spontaneous release was analyzed using the Mini Analysis Program (Synaptosoft Inc., Decatur, GA). Evoked EJP amplitude was corrected by using nonlinear summation (McLachlan and Martin, 1981; Feeney et al., 1998). The quantal content of evoked release was calculated from individual muscle by ratio of the averaged EJP and averaged mEJP amplitude. Statistical analyses of EJP and mEJPs between genotypes were made using Student's t test (SigmaPlot 10.0, Systat software Inc.).

FM1-43 dye uptake experiments were essentially performed as described (Verstreken et al., 2002). Wandering third instar larvae were dissected on Sylgard plates in HL-3 buffer without calcium and then incubated with 4 µM FM1-43 solution in modified HL-3 with high potassium (90 mM KCl, 25 mM NaCl, 10 mM NaHCO_3_, 5 mM HEPES, 30 mM Sucrose, 5 mM Threalose, 10 mM MgCl_2_, 1.5 mM CaCl_2_, pH = 7.2) for 60 seconds. Larvae were then washed five times for 2 mins in generous volumes of HL-3 without calcium. *syp* mutant and control larvae were tested in parallel on the same Sylgard plate and imaged immediately after washing.

### Transmission electron microscopy

*Drosophila* neuromuscular junction ultrastructure was imaged following standard Electron Microscopy procedures. Briefly, wandering third instar larvae were filleted and dissected at room temperature in 2.5 mM calcium HL-3 medium and subsequently fixed overnight in 2% paraformaldehyde/2.5% glutaraldehyde/0.1 M cacodylic acid (pH 7.2). The fixed fillets were then processed inside a Ted Pella Bio Wave microwave with the vacuum attachment. Samples were fixed again, followed by 3× water rinses, post-fixed with 1% aqueous osmium tetroxide, and followed again with 3 more rinses with Millipore water. A graded series of ethanol concentrations from 30–100% was used as the initial dehydrant followed with propylene oxide as a final dehydrant. Samples were gradually infiltrated with 3 propylene oxide and Embed 812 graded ratios into 3 changes of pure resin under vacuum. Samples were allowed to infiltrate in pure resin overnight on a rotator. The samples were embedded into flat silicone moulds and cured in the oven at 62°C for three days. The polymerized samples were sectioned and stained with 1% uranyl acetate for ten minutes followed by lead citrate for one minute before TEM examination. TEM images were captured using a JEOL JEM 1010 transmission electron microscope with an AMT XR-16 mid-mount 16 mega-pixel digital camera.

### Light microscopy

Live-cell imaging was performed on a custom-built upright widefield DeltaVision microscope (Applied Precision, Olympus IX70 with a Roper CoolSnap HQ) with water-immersion objectives (Olympus). Fixed material imaging was performed on a widefield DeltaVision microscope (Applied Precision, Olympus IX70 with a Photometrics EMCCD;Olympus objectives, 1.512 oil) except for larval central nervous systems which were image at on a confocal microscope system (Fluoview FV1000 IX81; Olympus) using a 60×/1.35 NA oil objective and FV1000 software (Olympus). Images are single confocal slices, or maximum or mean intensity projections of 25 z-stacks across 5 µm depth as indicated. All images were deconvolved using softWoRx (Applied Precision) (Parton and Davis, 2006) in order to re-assign out-of-focus light to the point of origin.

### Immunofluorescence and quantification of fluorescent images

Third instar larvae were size-matched and dissected according to standard protocols in HL-3 buffer with low calcium levels (1.5 mM) on Sylgard plates (Verstreken et al., 2008). Larvae were quickly washed three times in ice cold HL-3 buffer without calcium and then fixed in 4% paraformaldehyde in PBS with 0.1% Triton-X for 20 minutes (except for GBB staining where larvae were fixed in 4% paraformaldehyde in PBS with 0.01% Triton-X for 10 minutes). Larvae were typically washed in PBS 0.1% Triton-X for an hour, then blocked in PBS 0.1% Triton-X 0.1% NGS for four hours before incubation with primary antibody in the blocking solution overnight at 4°C with gentle rocking. Secondary antibodies with Alexa fluorophores (life technologies) were incubated for two hours at room temperature at 1/250 in PBS 0.1% Triton-X 0.1%. Larvae were mounted in ProLong Gold mounting medium and cured overnight at room temperature before being sealed with nail varnish. For quantifying NMJ structure ice cold HL-3 was used without calcium and with 1 mM EGTA to minimize spontaneous contraction during fixation. Primary antibodies used were Syp (McDermott et al., 2012; 1/300), P-MAD (Persson et al., 1998; 1/1000), GBB (Dani et al., 2012; 1/100), Wg (4D4 Developmental Studies Hybridoma Bank; 1/2), Brp (NC82 Developmental Studies Hybridoma Bank; fixed for 5 minutes in Bouin's Solution 1/100), GluR_II_C (fixed 5 minutes in Bouin's Solution; 1/1000), HRP (Jackson ImmunoResearch; 1/250). DAPI was incubated with larvae for 20 minutes at 1/1000. Perfect muscle morphology was maintained by incubating larvae in microcentrifuge tubes with flat bottoms (HydroLogix, Fischer) in which no more than five larvae were incubated at once. Larval central nervous system immunofluorescence was performed as in Daul et al. (Daul et al., 2010) and mounted in ProLong gold.

To quantify presynaptic fluorescence at the NMJ imaging conditions were standardized. DeltaVision files (.dv) were then processed using a bespoke macro for FIJI Image J. This macro generates a mask (i.e. a region of interest) based on marker fluorescence intensity using Otsu's automatic thresholding method. Average fluorescence intensity for the signal of interest in a second channel is calculated for all pixels that lie within the auto-thresholded mask, summing the contribution from each slice in the 3D stack. The macro was developed by Graeme Ball to allow automated analysis of voxel intensity within a 3D bouton structure labeled by anti-HRP fluorescence relative to background signal. A copy of the macro code is available as open source freeware; https://github.com/graemeball/ij_scripts/blob/master/Macros/Sum_Masked_Signal.ijm.

### Biochemistry

Five whole wandering third instar larvae were homogenised in 200 µl lysis buffer (10 mM Tris-HCl, 150 mM NaCl, 0.5 mM EDTA, 0.5% NP-40) and left on ice for 30 minutes. Lysates were standardised by Bradford Assay and heated to 95°C in 2× protein sample buffer (with reducing agent added prior to use) (Invitrogen) and loaded alongside a pre-stained standard (SeeBlue Plus2, Invitrogen) into NuPAGE 4–12% Bis-Tris Protein gel (NP0321PK2). Western blots were transferred onto nitrocellulose membranes and all antibodies were incubated and washed at room temperature in PBS 0.1% Tween-20 and 5% milk. Anti-Tubulin was used as a loading control (T9026 Sigma, 1/1000) to reveal a single 55 kDa band alongside Anti-Syp (McDermott et al., 2012; 1/3000). Quantitative, two-colour Western blot analysis was performed on three biological repeats using a LICOR Odyssey FC instrument and Image Studio V2.0 analysis software.

### Bioinformatics comparisons of Syp and related protein domains

Sequence similarities and percent identities were calculated between the three RRM domains 1–3 (and the alternative RRM1 present in isoform Syp-PC) of Syp and four closely related human proteins hnRNP Q3, hnRNP Q2, hnRNP Q1 and hnRNP R. RRM domains were located using SMART (Letunic et al., 2012). In all cases the pairwise Needleman–Wunsch algorithm (Needleman and Wunsch, 1970) was used to globally align domains, using the BLOSUM62 similarity matrix, a gap penalty of 10.0 and gap extension penalty of 0.5. Nuclear localization signals, monopartite and bipartate were mapped onto the Syp isoforms using NLS-Mapper (http://nls-mapper.iab.keio.ac.jp). RGG/RG motifs were mapped with a custom Perl script (available from http://www.darogan.co.uk) using the RGG/RG definitions from Thandapani et al. (Thandapani et al., 2013). These are situated in the same region of the protein as the canonical RGG/RG domains found in the protein sequences. Script writing and bioinformatic analysis was performed by Russell S. Hamilton.

## RESULTS

### Syp is required for vesicle release at the NMJ

**Fig. 1.**
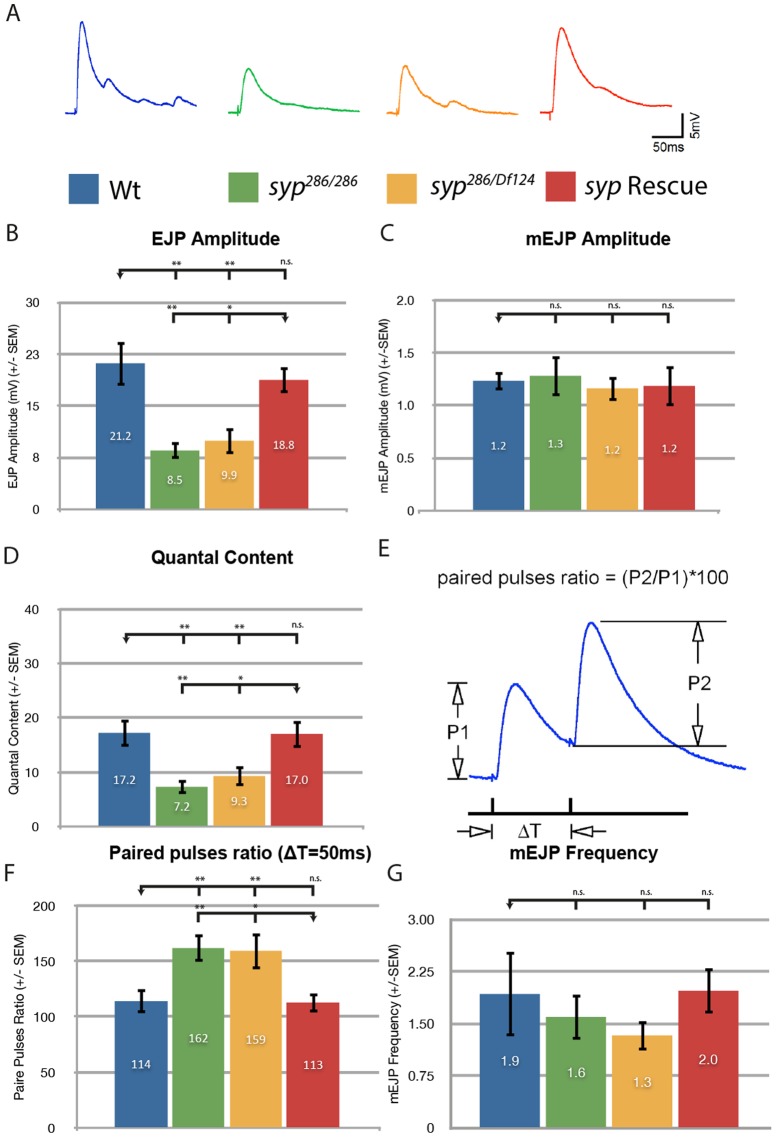
*syncrip* mutants exhibit a decrease in vesicle release probability. (A) EJP traces of EJP during 0.2 Hz of stimulation at 0.5 mM [Ca2+] at muscle A36 for wildtype (n = 6) *syp^286^/syp^286^* (n = 6) *syp^286^/syp^Df124^* (n = 6) and *syp Rescue* (n = 5). (B) The mean EJP amplitude is significantly reduced in syp mutants relative to controls (C) while the miniature(m)EJP amplitude and frequency (not shown) remain unchanged. (D) Accordingly the Quantal Content is significantly reduced in *syp* mutants relative to controls. (E,F) The paired-pulse ratio for a 50 ms time interval reveals that *syncrip* mutants exhibit a decrease in vesicle release probability. n refers to the number of animals tested. (G) mini (m) EJP frequency is unchanged in *syp* mutants. Independent two-tailed Student's t-test; *** p<0.001 ** p<0.005 * p<0.05, n.s. p>0.05.

Given that Syp is known to regulate the morphology of cultured neurons (Chen et al., 2012), we first tested whether Syp is required for synaptic output using an insertion in the *syp* gene (*syp^286^*) in combination with the deficiency Df(3R)BSC124 (*syp^Df124^*), both of which lack all isoforms of Syp protein and mRNA (McDermott et al., 2012). We performed electrophysiological analysis on muscle 6 of third instar larval body walls from *syp*-null larvae and recorded Excitatory Junction Potentials (EJP) in 0.5 mM Ca^2+^. We found that *syp* mutants exhibit a ∼50% decrease in EJP amplitude relative to wild type. A genomic construct containing a functional copy of Syp completely rescues the *syp* phenotype (Fig. 1A,B). In contrast, no difference in miniature EJP (m)EJP was recorded between control and test larvae (Fig. 1C). We therefore conclude that the number of vesicles released from the pre-synapse, or quantal content, is significantly diminished in *syp* mutants relative to controls (Fig. 1D). The quantal content is proportional to both the number of vesicle release sites, or Active Zones (AZs), and the probability of release. Interestingly, there is no change in the distribution or levels of the AZ marker Bruchpilot (Brp), nor are postsynaptic glutamate receptor complexes densities altered per bouton relative to controls (supplementary material Fig. S1A–C). We have also shown in a parallel study that the number of boutons innervating muscles 6 and 7 are increased in *syp* mutants relative to controls (McDermott et al., 2014). Taken together with the wildtype mEJPs amplitudes recorded, we conclude that *syp* mutants form functional synapses and the deficit in EJP recorded is not attributable to decreased expression of presynaptic AZs or postsynaptic receptor complexes. Our electrophysiological data therefore suggest that the probability of vesicle release is diminished in *syp* mutants.

To test more directly whether the probability of vesicle release is diminished in *syp* mutants, we tested whether unreleased vesicles can be detected accumulating at AZs following an action potential. We used paired-pulse stimulation (PPS) to determine whether a second pulse, 50 ms after a first pulse, evokes a greater potential in *syp* mutants compared to controls (Fig. 1E). We found that *syp* mutants exhibit a large increase in the paired pulses ratio relative to wildtype, a phenotype that is completely rescued by the *syp* genomic rescue construct (Fig. 1F). As PPS is considered to be a function of presynaptic vesicle release (Futai et al., 2007; Giagtzoglou et al., 2009), we conclude that *syp* mutants have a decreased probability of pre-synaptic vesicle release.

### Syp is required for synaptic vesicle metabolism and the structure of the synapse

**Fig. 2.**
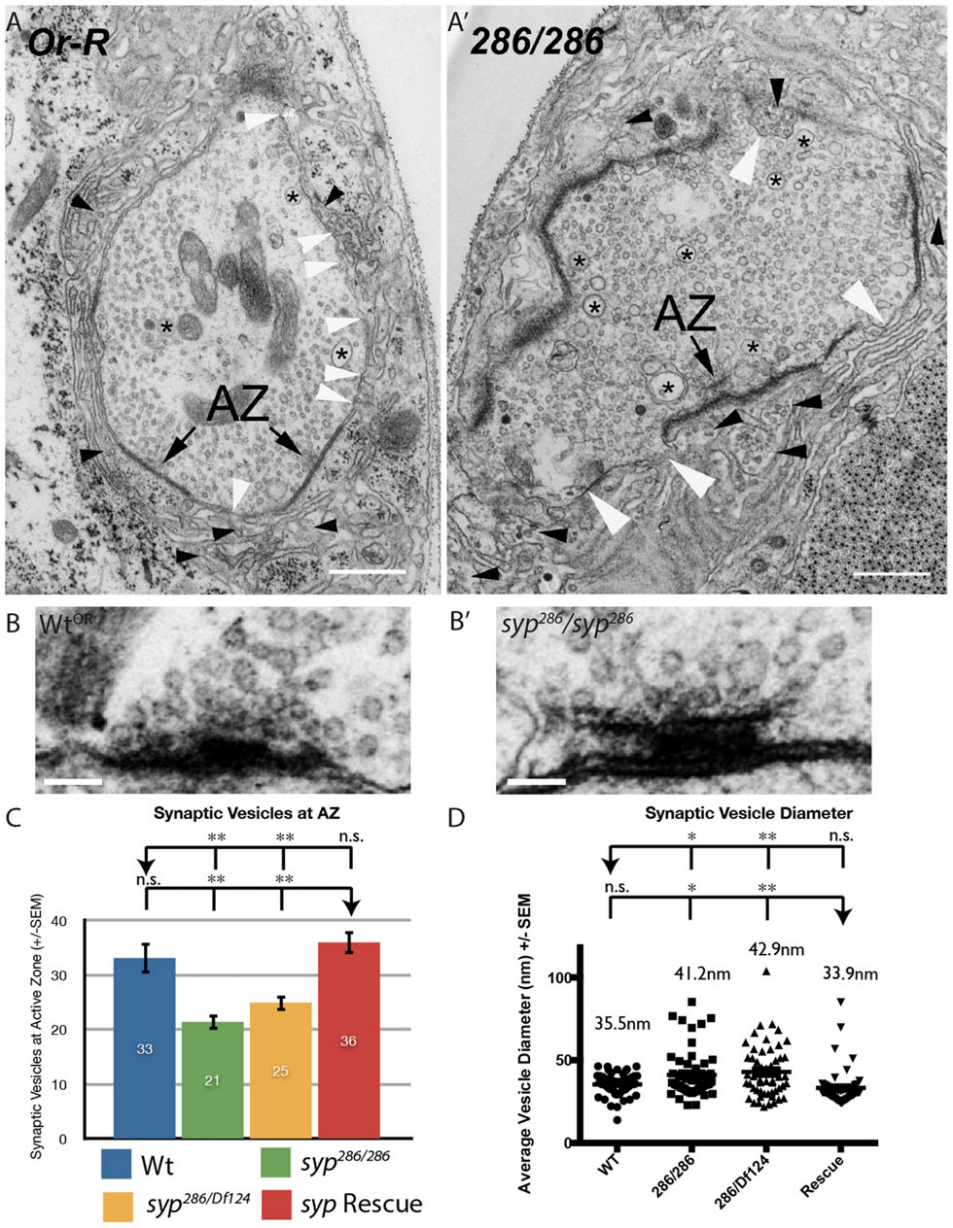
*Syncrip* mutants exhibit defects in synapse structure and vesicle docking. (A,A′) Ultrastructure analysis of *syncrip* mutant synapses reveals dramatic defects in synapse structure and organization. *syp* mutant boutons contain a larger population of large vesicles (asterisk) than wildtype and exhibit multiple points at which the pre- and postsynaptic membrane are not apposed (white arrowheads). While lesions are present in the wildtype boutons also, they are much more prevalent in *syp* mutants. In *syp* mutants large clusters of synaptic vesicles are present in the postsynapse (black arrow heads), though this likely is caused by fixation and sectioning and reflects a weakness in synaptic architecture relative to wildtype (see main text). (B–C) Consistent with a deficit in vesicle release, *syp* mutants have fewer vesicles docked, or close to, active zones relative to controls. Vesicles were counted within a 500 nm × 25 nm area around active zones in wt (n = 7), *syp^286^/syp^286^* (n = 14), *syp^286^/syp^Df124^* (n = 25), *syp Rescue* (n = 13). The wildtype T-bar was chosen as it is representative of the number of docked vesicles observed. *syp* mutant terminals contained enlarged T-bars. (D) *syncrip* mutants exhibit increased vesicle size (n>30 vesicles counted across ≥3 boutons for each test). Scale bars: (A,A′) 500 nm, (B,B′) 100 nm. Independent two-tailed Student's t-test; *** p<0.001 ** p<0.005 * p<0.05, n.s. p>0.05.

Many defects in vesicle release are known to be caused by misorganisation of the synapse. To gain insight into how Syp may regulate the probability of vesicle secretion, we performed transmission electron microscopy on third instar larval junctions. Wildtype synapses are characterized by the docking of uniform synaptic vesicles at discrete AZ structures, and the close apposition of neuronal and muscle membrane. We found that *syp* mutants exhibit a number of defects in this organisation (Fig. 2A,B). Most prominently, *syp* mutant boutons contain a heterogenous population of vesicles with a larger average size relative to wildtype, including a small population of exceptionally large vesicles that were over 100 nm in diameter (Fig. 2A,B, asterisks). While the mean size of vesicles was restored to near wildtype levels in the rescue animal (Fig. 2D), a few large vesicles were still present in the rescue boutons.

One possible cause of the abnormally-sized vesicles in *syp* mutants is that Syp is required for proper endocytosis and vesicle formation. To assay for endocytosis we applied 90 mM K^+^ and 5 mM Ca^2+^ to stimulate the NMJ and measured the uptake of the styryl dye FM1-43 (Verstreken et al., 2005). While *syp* mutants exhibit clear deficits in FM1-43 fluorescence relative to wildtype controls (supplementary material Fig. S2F,G), this is not fully restored in the genomic rescue. Given that the genomic rescue line completely restores all other mutant phenotypes tested, and that *syp* mutant terminals exhibit no obvious decrease in vesicles, it is probable that Syp is not required for endocytosis, but that *syp* mutants exhibit a decrease in FM1-43 fluorescence for another reason, such as subtle defects in vesicle trafficking reported in *drp1* mutants (Verstreken et al., 2005). Another possible explanation is that the genomic rescue expresses only ∼30% of the wildtype levels of Syp, and this protein level is sufficient to rescue most, but not all, aspects of the mutant phenotype (supplementary material Fig. S3). While the lack of rescue does not exclude defects in endocytosis, it is not possible to reliably conclude whether Syp is required for proper endocytosis from these data.

Paired-pulses stimulation revealed that *syp* mutants exhibit a decreased probability of vesicle release. This prompted us to test whether synaptic vesicles were appropriately docked at active zones in *syp* mutants. Systematic analysis reveals that fewer vesicles are found docked at, or in close proximity to (within a 500 nm by 25 nm area), active zones in *syp* mutants, despite the enlarged size of AZs in *syp* mutants. This may underlie the recorded deficit in vesicle release probability (Fig. 2B,C). The defects in docking observed in *syp* mutants are absent from wildtype and genomic rescue controls. The EM ultrastructure also revealed that the Post Synaptic Density (PSD) are significantly enlarged in *syp* mutants relative to wildtype and genomic rescue controls, possibly reflecting increased postsynaptic protein production (supplementary material Fig. S2). We conclude that Syp is required for proper vesicle biogenesis and docking at active zones, and that *syp* mutants exhibit enlarged AZs and PSDs.

We also observed lesions across the synaptic membrane of *syp* mutants, leading to vesicles spilling into the Subsynaptic Reticulum (SSR; Fig. 2A,B). While we observe small lesions in wildtype controls (white arrowheads), and rare postsynaptic vesicles (black arrowheads), these are dramatically more frequent in *syp* mutants. We were unable, however, to detect synaptic vesicles markers in the SSR of *syp* mutants via immunofluorescence or live cell imaging (Cysteine String Protein and Synaptotagmin 1-GFP; data not shown). Instead, we conclude that the synaptic membranes are weakened in the absence of Syp, and that fixation for TEM leads to tears across the synapse. Taken together these data highlight roles for Syp in regulating synaptic vesicle docking in the pre-synapse, and maintaining the robust integrity of the synaptic membrane.

### Syp is expressed in the larval brain and muscle, but is not detectable in motoneurons

**Fig. 3.**
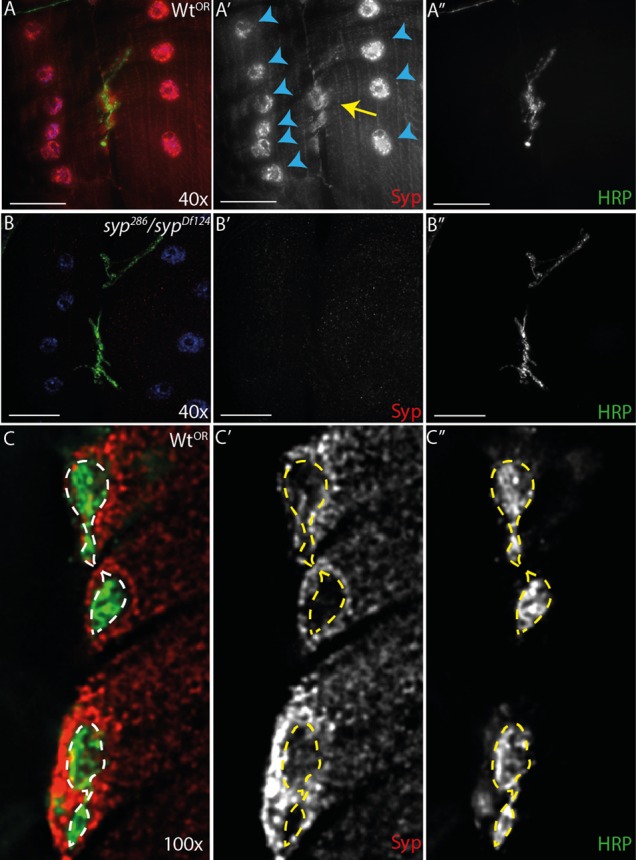
Syncrip is enriched in muscle nuclei and at the postsynapse, but is undetectable at the presynapse. (A–A″) Sensitive wide-field imaging coupled with a polycolonal antibody reveals Syncrip throughout the muscle cytoplasm, with enrichment in the nuclei (blue arrowheads) and at the postsynapse (yellow arrow). (B–B″) The Syncrip antibody is highly specific and registers little signal in the *syp* mutants. (C–C″) Higher magnification imaging fails to robustly detect Syncrip in the presynapse above background fluorescence. Images are maximum intensity 5 µm projections. Scale bars: (A–B″) 40 µm, (C–C″) 5 µm.

To test whether the vesicle release defects we observe in *syp* mutants are due to Syp's function in the motoneuron, we studied the distribution of the protein in third instar larvae using a polyclonal antibody against Syp (McDermott et al., 2012). Consistent with other studies, we found that Syp is expressed throughout the muscle cells, is enriched in muscle nuclei and at the NMJ post-synaptic terminals (McDermott et al., 2014). Surprisingly, Syp could not be detected in pre-synaptic terminals (Fig. 3A,C), nor in the motoneuron axon bundles (data not shown). In control experiments, we found that Syp is completely absent from *syp* null mutants (Fig. 3B). These results could either be due to a genuine presence of Syp in the SSR, or alternatively to non-specific binding of the antibody to the SSR and a lack of SSR in the *syp* mutant. To distinguish between these possibilities, we examined the SSR in *syp* mutants. We found that the SSR marker Discs Large robustly stained the postsynapse in *syp* mutants, and that there was no change in SSR width between *syp* mutants and controls (supplementary material Fig. S2E). Therefore, we conclude that the immunofluorescent signal detected at the postsynapse represents genuine Syp antigen, rather than non-specific binding at the SSR.

**Fig. 4.**
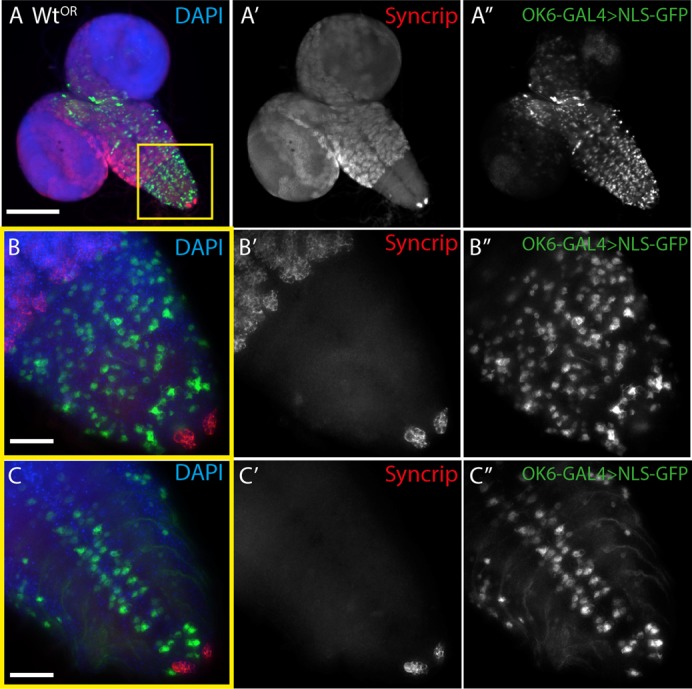
Syncrip is expressed in the larval central nervous system, but it not detectable in motoneurons. (A–A″) Syncrip is found in multiple cells of the third instar central nervous system resembling neuroblastomeres but was largely absent from the ventral nerve cord. (B–B″) Expression of nls-GFP by the motoneuron-specific driver OK6-GAL4 reveals that Syncrip is not detectable in motoneurons. (C–C″) Cross-section through ventral nerve cord centre. Images are single confocal slices. Scale bars: (A–A″) 40 µm, (B–C″) 10 µm.

To investigate whether Syp is present in the motoneuron cell bodies, we examined Syp immunofluorescence in the larval central nervous system (Fig. 4). Consistent with our previous studies we found that Syp was expressed in many cells in the larval brain, including the optic lobe medullary neuroblastomeres and thoracic lineages that resemble neuroblastomeres (McDermott et al., 2012; Kuzin et al., 2012). Strikingly, Syp was not detected in the motoneurons in the ventral nerve cord midline. To visualize motoneurons, GFP conjugated to a nuclear localization signal was expressed in the larval CNS using the motoneuron driver OK6-GAL4 (Fig. 4B,C). Immunofluorescent staining of Syp in larvae expressing GFP in motoneurons revealed no detectable expression of Syp in the motoneurons cell bodies. It is likely that the Syp antibody is penetrating the CNS tissue as we able to detect other proteins in the motoneurons using the same fixation and staining conditions (data not shown). We also tested the expression levels of Syp using different RNAi lines and driver combinations by quantitative Western Blots from central nervous tissue only. When the anti-Syp RNAi constructs were driven in all neurons by ELAV-GAL4, Syp expression was reduced by ∼90%. However when RNAi was driven by OK6-GAL4 in motoneurons alone, no reduction in Syp could be detected in the central nervous system (supplementary material Fig. S5A,B). Taken together, these data suggest that Syp is expressed in the postsynaptic compartment, but not in the presynaptic compartment. Nevertheless, it is not possible to exclude the possibility that Syp is expressed in the motoneuron below the threshold for detection, or at an earlier developmental stage. Interestingly, expression of Syp in muscle is consistent with the distribution of mammalian SYNCRIP, which is found in RNP particles in post-synaptic dendrites (Bannai et al., 2004; Duning et al., 2008). Taken together, our results suggest that Syp acts non-autonomously in the muscle to regulate the neuromuscular junction presynaptically.

### Syp regulates the levels of retrograde signaling molecules

**Fig. 5.**
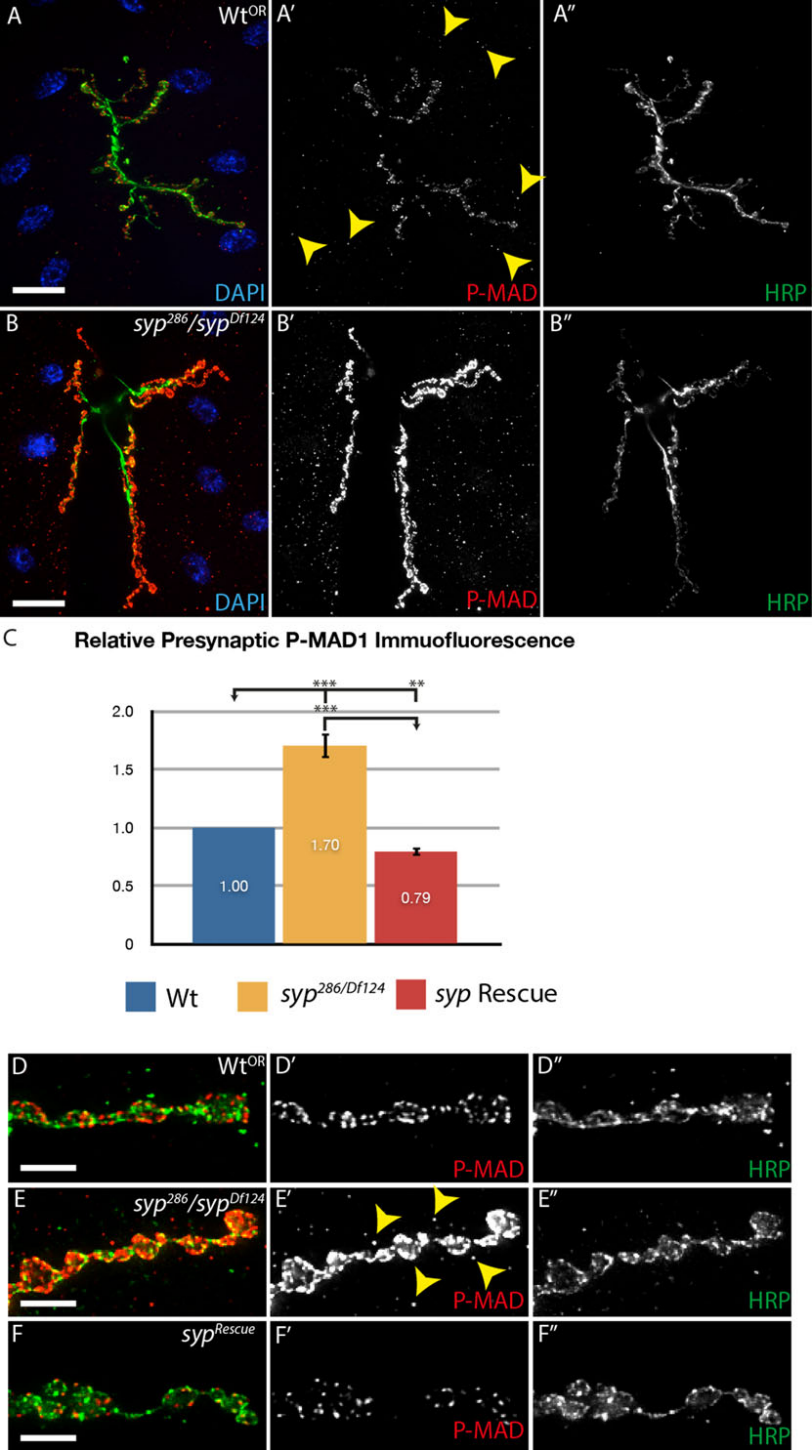
*syncrip* mutants exhibit elevated P-MAD levels in both muscle and neuron. (A,B) Merged image showing DAPI stained muscle nuclei (blue), motoneurons bouton stained with HRP (green) and P-MAD signal (red). P-MAD was elevated in both the presynaptic and postsynaptic compartment of *syncrip* mutants relative to controls. (A′,B′) Single channel showing P-MAD staining. (A″,B″) Single channel showing HRP staining. (A–A″) wild type. (B–B″) *syp* mutant (syp^286^/Df^124^). Yellow arrowheads in A′ indicate low levels of postsynaptic P-MAD in wild type controls. (C) Bespoke imaging quantification tools reveal that *syp* mutants exhibit a ∼1.7-fold increase in the presynaptic levels of phosphorylated MAD relative to wildtype, indicating upregulated retrograde signaling. Quantification of the P-MAD immunofluorescence was performed on full z-stacks. Independent two-tailed Student's t-test; *** p<0.001, ** p<0.005 (* p<0.05, n.s. p>0.05). (D–F) Merged high magnification maximum intensity projections of 5 µm thickness showing HRP (green) and P-MAD (red). (D–D″) wild type. (E–E″) *syp* mutant (syp^286^/Df^124^). (F–F″) rescued *syp* mutant. (D′–F′) Single channel showing P-MAD staining. (D″–F″) Single channel showing HRP. Scale bars: (A–B″) 40 µm, (D–F″) 5 µm.

An obvious explanation for the non-autonomy of Syp at the NMJ would be if Syp affects retrograde signaling from the muscle to the synapse. We therefore tested the hypothesis that Syp may regulate BMP retrograde signaling, one of the most well-studied retrograde signaling pathways (Keshishian and Kim, 2004). We used an antibody that recognizes only the phosphorylated form of MAD (P-MAD), an established method to quantify the output of BMP signaling from muscle to pre-synapse (Persson et al., 1998; McCabe et al., 2003; Dani et al., 2012). We used bespoke image analysis software to quantify the levels of P-MAD in *syp* mutants versus control larvae in both neuron and muscle, as P-MAD is known to be present in both cells (Dani et al., 2012). We found that *syp* mutants exhibit a significant increase in the levels P-MAD in both cell types, with a ∼1.7-fold increase in the presynaptic compartment, compared with wild type and genomic rescue controls (Fig. 5). We therefore conclude that Syp is required to regulate presynaptic P-MAD.

**Fig. 6.**
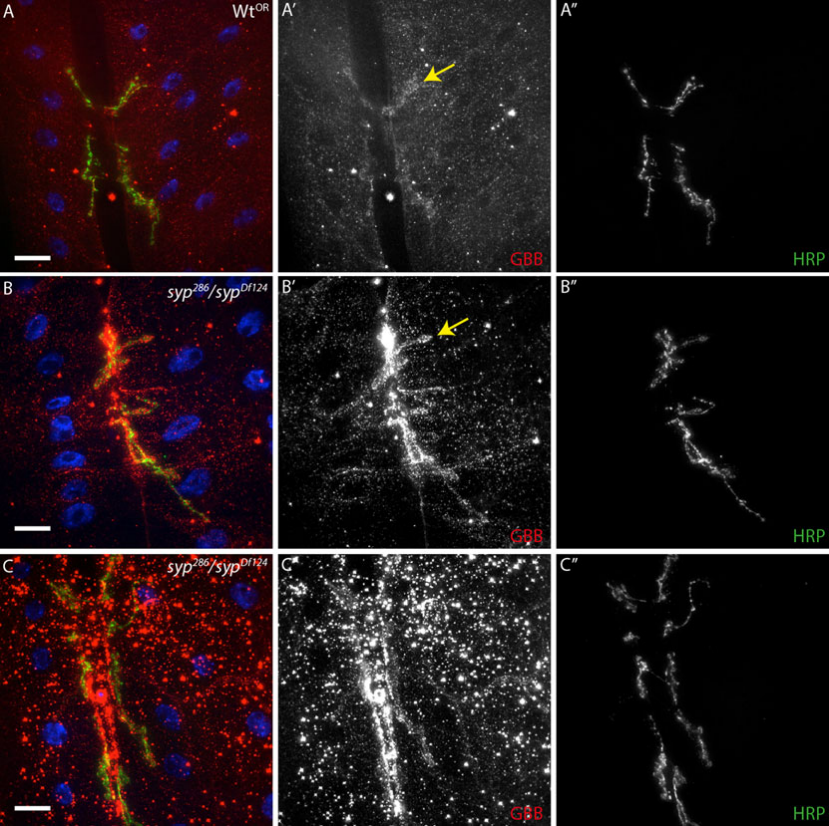
Glass Bottom Boat (GBB) levels are increased throughout the muscle, and at the postsynapse (arrow), of *syncrip* mutants. (A–C″) Anti-GBB immunofluorescence in syncrip mutants is dramatically elevated throughout the muscle and at the postsynapse relative to wildtype controls, indicating that Syncrip regulates BMP retrograde signalling through GBB. Images are maximum intensity 5 µm projections (quantitative analysis performed on full z-stack). Scale bars: (A–B″) 40 µm, (C–C″) 5 µm.

Phosphorylation of MAD in the pre-synapse is increased in response to secretion of the GBB ligand from the muscle to the neuron (McCabe et al., 2003). As Syp is expressed in the muscle, we hypothesised that GBB signaling would be elevated in *syp* mutants. To test this, the levels and distribution of GBB were examined in *syp* mutants using a polyclonal anti-GBB antibody (Dani et al., 2012). We found that *syp* mutants exhibit a very significant increase in GBB levels, both throughout the muscle and post-synaptically, relative to wildtype (Fig. 6A–C). In some cases GBB is present in large foci within the *syp* muscle cytoplasm (Fig. 6C). Technical limitations prevented us from testing whether the excess GBB observed in *syp* mutants was secreted properly, or whether the genomic rescue could restore this phenotype. Given the correlation between enhanced GBB signalling in the muscle, and P-MAD in the neuron, we conclude that Syp is likely required to suppress GBB protein levels and signaling in the muscle.

GBB signaling occurs from muscle to neuron. In parallel, signaling in the reverse direction occurs via the trans-synaptic signaling molecule Wingless (Packard et al., 2002). To test whether a loss of Syp lead to a global increase in signaling across the synapse we examined the levels of Wingless (Wg) in *syp* mutants. We found no appreciable difference in the levels or distribution of Wg between *syp* mutants and controls (supplementary material Fig. S4A,B). Thus, while *syp* mutants exhibit increased signaling from muscle to neuron, it appears that Syp is not required for transsynaptic signaling from neuron to muscle. Taken together, these data indicate that expression of the BMP ligand is specifically upregulated in *syp* mutants, with hyperactivation of the downstream transcription factor MAD. We conclude that Syp is required to regulate neuronal synapse structure and function, and signaling through the Bone Morphongic Protein pathway.

## DISCUSSION

### Syp links postsynaptic translation with retrograde signaling

Our results highlight the conserved mRNA-binding protein Syp as a novel factor required in the post-synapse for the modulation of synaptic output in the pre-synapse. During synaptic plasticity, neurotransmitter released from the pre-synapse is thought to signal changes in protein production at the post-synapse, which modulates the structure and efficacy of the post-synapse. In the reverse direction, retrograde signals from the postsynapse are thought to signal back to regulate the structure and the rate of secretion from the pre-synapse. Despite their importance, little is known about how these two processes are balanced and coordinated across the synapse during development and synaptic plasticity, which is crucial for memory and learning. We have identified the RNA binding protein Syp as a new factor that influences both these processes. Given that the mammalian homologues of *Drosophila* syp (SYNCRIP/hnRNP Q1 and 2) restricts translation in both neuronal and non-neuronal cells (Chen et al., 2012; Svitkin et al., 2013), we propose a model in which Syp co-ordinates translation in the post-synapse with retrograde signaling to the pre-synapse, thus fine-tuning both sides of the synapse. In support of this model, we find that loss of Syp leads to an upregulation of retrograde signaling factors. *syp* mutants also show enlarged post synaptic densities and a dramatic decrease in the rate of presynaptic vesicle release. In addition, in a parallel study we have revealed that Syp is genetically required only in the muscle to regulate NMJ morphology (McDermott et al., 2014), since loss of Syp leads to synapse overgrowth and over expression of Syp leads to synapse undergrowth. While we cannot completely exclude the possibility that Syp also has a role in the motoneuron, this seems unlikely. First, we cannot detect Syp protein in the motoneuron and second, RNAi depletion experiments show that Syp is only required postsynaptically and not presynaptically for correct NMJ morphology.

Previous genetic studies have revealed a well characterised pathway for GBB retrograde signaling. Loss of Glass Bottom Boat leads to NMJ undergrowth, defects in the integrity of the synaptic membrane, vesicle size, and a decrease in synaptic output, and many of these phenotypes are rescued by expression of GBB expression in muscle (McCabe et al., 2003). Accordingly, loss of the presynaptic GBB receptors, Wishful thinking and Thick veins, and the downstream R-smads, also lead to similar phenotypes (Aberle et al., 2002; Rawson et al., 2003; McCabe et al., 2004). Together these studies show that a decreased in GBB signalling leads to synapse undergrowth and decrease in synaptic output. Our results support a role for Syp in lowering GBB retrograde signalling as we have found that in *syp* mutants GBB protein is elevated and the NMJ synapse is overgrown. However, one aspect of the syp phenotype that does not fit this model is a reduction in the efficiency of vesicle release, which is the opposite of that expected from an increase in GBB signaling. There are several possible explanations to this discrepancy. While the phenotype of GBB loss of function has been characterised in detail, to our knowledge over expression of GBB has only been used to rescue mutant phenotypes (McCabe et al., 2003; McCabe et al., 2004; Goold and Davis, 2007). It is therefore possible that over expression of GBB and P-MAD leads to similar effects on vesicle release as the loss of GBB function. Consistent with this possibility, disruption of the ubiquitin ligase Highwire leads to upregulation of BMP signalling, and to a decrease in synaptic transmission and NMJ overgrowth similar to *syncrip* mutants (Wan et al., 2000; Wu et al., 2005; McDermott et al., 2014). Another possibility is that syp could also be influencing the pre-synapse through roles in other cell types. Supporting this idea is the fact that Syp is present and required in many tissues, has many downstream targets (McDermott et al., 2014) and its mechanism of action is likely to be different in different individual targets and tissues. Syp could therefore influence vesicle release efficiency in the motorneurons through a role in glia or interneurons. Finally, in a parallel study, we performed immunoprecipitation of Syp followed by high-throughput sequencing to assess the RNA binding partners of Syp (McDermott et al., 2014). Syp associates with multiple transcripts encoding key synaptic regulators, as well as many mRNAs encoding proteins of unknown function. While Syp does not associate with *glass bottom boat* mRNA, it is quite possible that it regulates the BMP pathway by binding an mRNA encoding one of the pathway's many other components, a novel BMP regulator, or indeed a previously undiscovered parallel retrograde signalling pathway.

Adding to the complexity of Syp's function, both mammalian SYNCRIP and *Drosophila* Syp contain three canonical RNA binding domains, RNA recognition motifs (RRMs), which are known to sometimes bind proteins as well as RNA (Maris et al., 2005) and mammalian SYNCRIP contains an additional RGG/RG rich C-terminal domain that is likely to interact with proteins. This domain is thought to promote interaction between mammalian SYNCRIP and multiple Synaptotagmins *in vitro* through their common C_2_B domain (Mizutani et al., 2000). While there are 15 known vertebrate Synaptotagmins, only two (Syt 4 and 7) are expressed in the *Drosophila* third instar larval muscle (Adolfsen et al., 2004). Intriguingly, vesicle trafficking by Synaptotagmin 4 at the postsynapse has been implicated in retrograde signaling in both flies and mammals (Yoshihara et al., 2005; Dean et al., 2009), while the role of Syt 7 is less clear. As a result, Synaptotagmin 4 may seem an attractive candidate to interact with *Drosophila* Syp. Inspection of the *syp* gene however shows that *Drosophila* Syp lacks the 161 amino acid C-terminal domain found in mammalian SYNCRIP that is both necessary and sufficient for association with the Synaptotagmin C_2_B domain (supplementary material Fig. S6) (Mizutani et al., 2000). The Synaptotagmin-interacting domain in mammalian SYNCRIP is rich in RGG/RG motifs, which can facilitate protein–protein interaction (reviewed by Thandapani et al., 2013). However, while Syp's RRM domains are highly conserved between mammals and flies, *Drosophila* Syp contains no canonical RGG/RG motifs. Moreover, genome-wide searches for canonical RGG/RG motifs in flies and mammals detect mammalian SYNCRIP/hnRNP Q and R, but not *Drosophila* Syp. It is therefore unlikely that Syp mediates retrograde signaling through interaction with Synaptotagmins, though further analysis is required to rule this out conclusively.

Interestingly, mammalian SYNCRIP has been shown to bind polyA sequences and interact with polyA binding protein (PABP) (Svitkin et al., 2013). PABP interacts with a wide variety of different complexes, including the microRNA-induced silencing complex (miRISC) (Moretti et al., 2012) and mRNA deadenylating factors (Fabian et al., 2009), to serve as a key regulator of global translation. Moreover, PABP has been shown to accumulate at the NMJ post-synapse to promote experience-dependent local translation that alters the efficacy of the synapse (Sigrist et al., 2000). Owing to the enrichment of Syp at the NMJ post-synapse, it is possible that Syp regulates the local expression of key members of the BMP pathway through interaction with the poly(A) tail. However, other studies have revealed Syp discriminates between different mRNAs and associates with specific transcripts (McDermott et al., 2012; McDermott et al., 2014; Chen et al., 2012). As loss of Syp leads to enlargement of the post synaptic densities, Syp may act to restrict translation of specific mRNAs encoding key synaptic proteins. The precise mechanism through which Syp binds specific mRNAs is unknown, but a greater understanding of the *in vivo* mRNA targets of Syp is likely to reveal common sequences or RNA structures, as well as identifying potential key regulatory targets for localised translation.

In linking retrograde signaling to translation in the postsynaptic compartment, Syp fits with a growing body of evidence showing a role for RNA-binding proteins in integrating RNA metabolism with other key cellular processes (Castello et al., 2012). Through interaction with multiple mRNAs and possibly protein targets, Syp could integrate a number of different synaptic processes. Indeed, we propose that there are clear advantages for integrating trans-synaptic signaling with postsynaptic translation to balance output on both sides of the synapse. It is interesting to consider that other RBPs central to neurobiology may also be “moonlighting” in seemingly unrelated processes. This diverse repertoire of RBP functions may explain why mutations in RBPs often lead to neurodegenerative diseases with complex phenotypes (Hanson et al., 2012). One such highly complex disease phenotype is caused by Fragile X mental retardation protein (FMRP), which interacts with many mRNAs directly. Interestingly, recent work has also revealed that Syp and FMRP are present in the same mRNP granule (Chen et al., 2012), although they do not interact directly. Furthermore, separate studies have revealed that SYNCRIP binds with wildtype Survival of Motor Neuron (SMN) protein, but not the truncated or mutants forms found in Spinal Muscular Atrophy (Rossoll et al., 2002), and Syp genetically interacts with *Smn* mutations *in vivo* (Sen et al., 2013). While the functional significance of Syp's diverse interactions is not yet fully clear, such data highlight that Syp, like many other RBPs, has an elaborate set of molecular interactions that lead to a complex phenotype when the gene is mutated.

While multiple studies have detected SYNCRIP in the dendrites of mammalian neurons, and experiments in cell culture have revealed the protein as a regulator of translation, the exact *in vivo* role of SYNCRIP remains untested in mammals. Our work highlights a new role for Syp in regulating synapse function and BMP signaling in *Drosophila*. Given the high degree of similarity between *Drosophila* and mammalian Syp, the importance of BMP signaling in multiple human diseases (Bandyopadhyay et al., 2013) and the well conserved features of the *Drosophila* NMJ as an *in vivo* model to investigate BMP pathways (Bayat et al., 2011), it seems likely that Mammalian SYNCRIP will also be shown to have important function in BMP signaling and synapse biology.

## Supplementary Material

Supplementary Material
