# Supplementary material for: Syncrip/hnRNP Q influences synaptic transmission and regulates BMP signaling at the Drosophila neuromuscular synapse
Source: Biol Open. 2014 Aug 29;3(9):839–49. doi: 10.1242/bio.20149027 (PMC4163661; doi:10.1242/bio.20149027)
Supplement: Supplementary Material [file supp_3_9_839__index.html]

Syncrip/hnRNP Q influences synaptic transmission and regulates BMP signaling at the Drosophila neuromuscular synapse — Syncrip/hnRNP Q influences synaptic transmission and regulates BMP signaling at the Drosophila neuromuscular synapse — Supplementary Material 

# Syncrip/hnRNP Q influences synaptic transmission and regulates BMP signaling at the *Drosophila* neuromuscular synapse

## bio.20149027 Supplementary Material

**Files in this Data Supplement:**

- Supplementary Material - James M. Halstead et al. doi: 10.1242/bio.20149027
