# Supplementary material for: Syncrip/hnRNP Q influences synaptic transmission and regulates BMP signaling at the Drosophila neuromuscular synapse
Source: Biol Open. 2014 Aug 29;3(9):839–49. doi: 10.1242/bio.20149027 (PMC4163661; doi:10.1242/bio.20149027)
Supplement: Supplementary Material [file supp_bio.20149027_bio.20149027-s1.pdf]

Biology Open (2014) 000, 1–11 doi:10.1242/bio.20149027

Supplementary Material
James M. Halstead et al. doi: 10.1242/bio.20149027

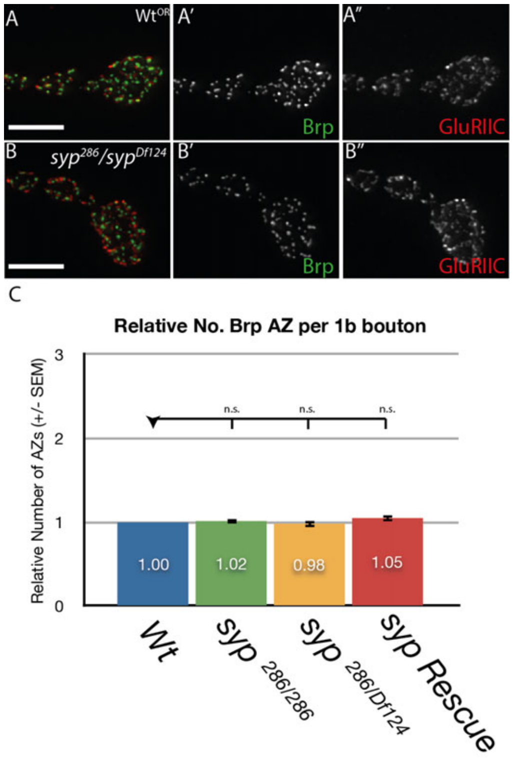

Fig. S1. ***syp*** **mutant boutons exhibit a wildtype number of Active Zones appropriately apposed to glutamate receptor complexes.** (A) *syncrip* mutants exhibit no change in the apposition of Active Zones (marked by Bruchpilot) or glutamate receptors (marked by GluRIIC). (B) The number of Active Zones are similar in *syncrip* mutants relative to controls. Images are maximum intensity 5 µm projections (quantitative analysis performed on full z-stack). Scale bars: 5 µm. Independent two-tailed Student's *t*-test; n.s. $p>0.05$.

Biology Open

Biology Open (2014) 000, 1–11 doi:10.1242/bio.20149027

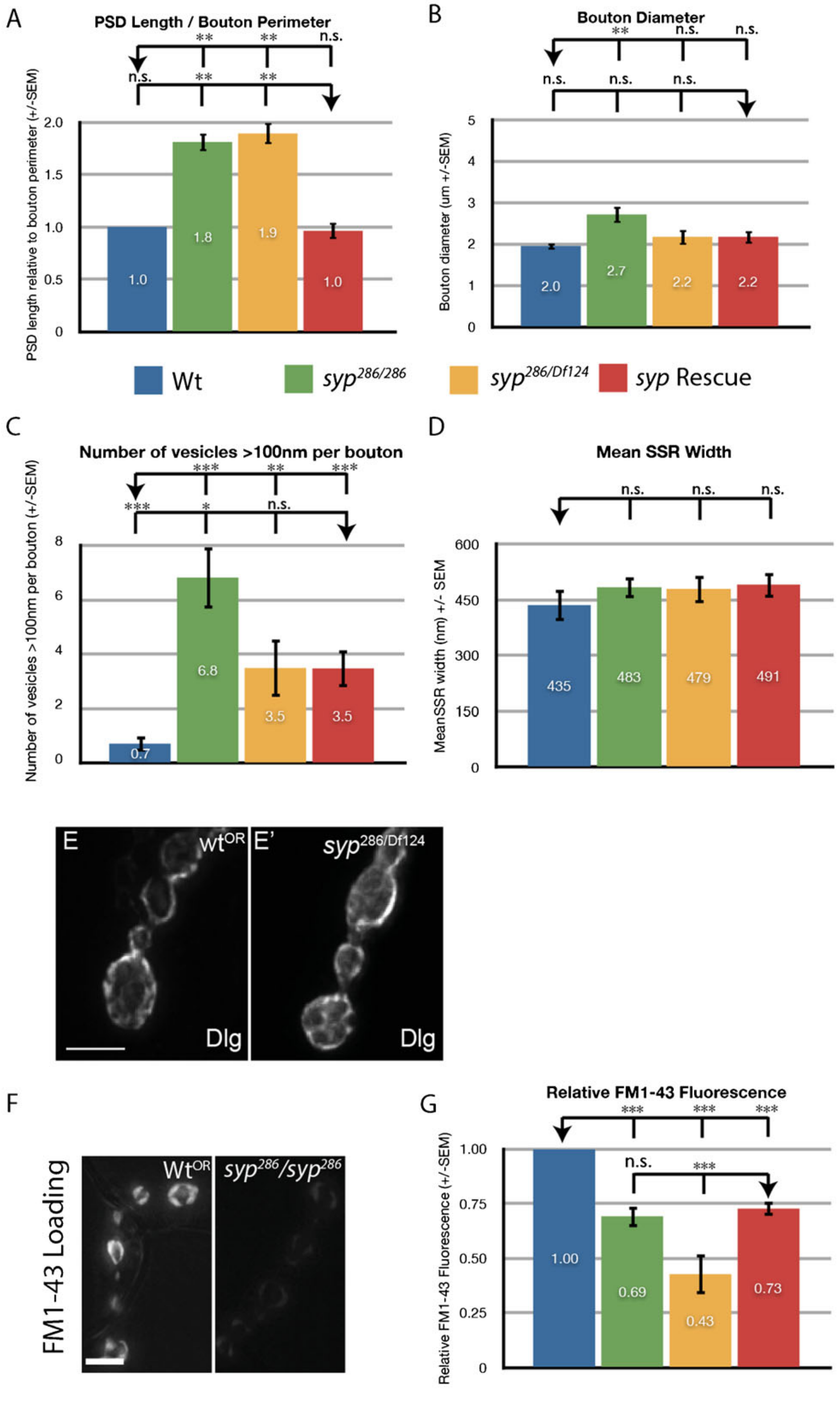

Fig. S2. Quantified analysis of synapse structure in *syncrip* mutants. (A) *syncrip* mutant boutons exhibit increased PSD length relative to controls, while (B) bouton diameter remains unchanged. (C) *syncrip* mutants contain a small number of exceptionally large vesicles that are also present in the genomic rescue line. (D) The width of the subsynaptic reticulum (SSR), and (E,E′) immunofluorescence of the SSR marker Discs Large (Dlg) is unchanged in *syp* mutants relative to controls. (F,G) *syp* synapses show reduced uptake of the fluorescent dye FM1-43 following 5 minutes of 90 mM K+, though as the Rescue construct does not restore this phenotype, it is not clear if Syncrip is required for endocytosis. Scale bars: (E,E′,F) 5 µm. Independent two-tailed Student's t-test; *** $p<0.001$ ** $p<0.005$ * $p<0.05$, n.s. $p>0.05$.

Biology Open

Biology Open (2014) 000, 1–11 doi:10.1242/bio.20149027

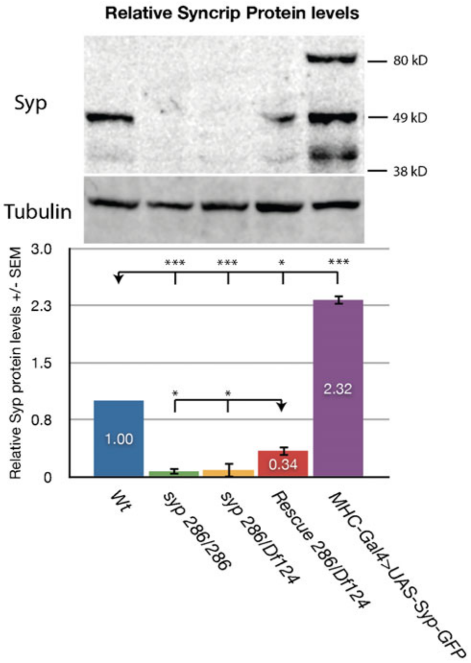

Fig. S3. Relative levels of Syncrip expression in *syncrip* alleles and controls. Quantitative Western blots reveal the syncrip mutants to be protein nulls and the genomic rescue to express ~34% Syncrip relative to wildtype. Quantification performed on the ~50 kDa Syncrip band. Independent two-tailed Student's t-test; p<0.001 ** p<0.005 * p<0.05, n.s. p>0.05.

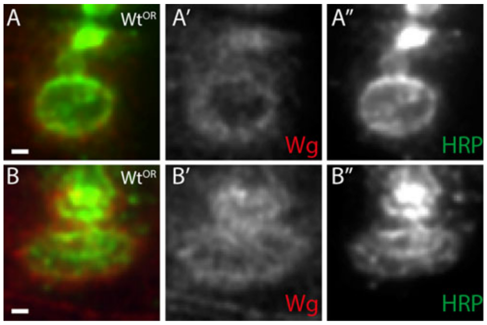

Fig. S4. Wingless signaling is unaffected in *syncrip* mutants. (A–B″) Wingless levels are similar in *syncrip* mutants and wildtype controls indicating that Syncrip regulates retrograde, but not anterograde signaling across the synapse. Images are maximum intensity 5 µm projections. Scale bars: 1 µm.

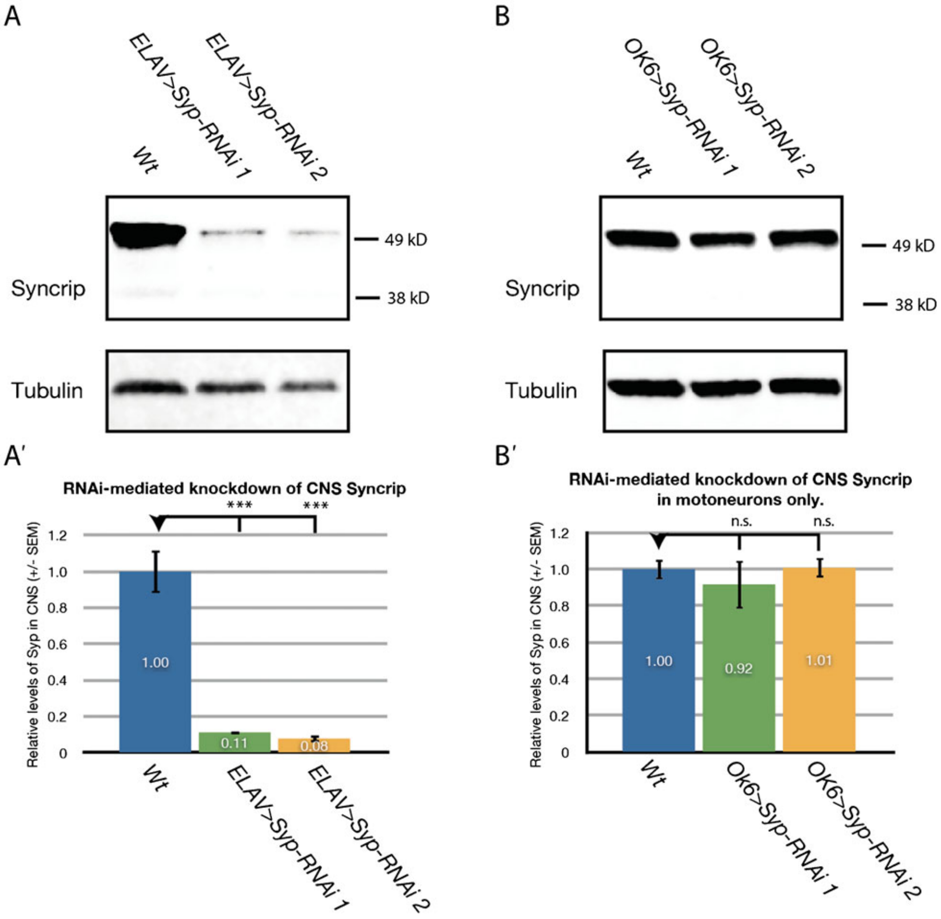

Fig. S5. RNAi-mediated knockdown of Syncrip cannot be detected in motoneurons. Relative levels of Syncrip expression from third instar larvae central nervous tissue where two RNAi constructs were expressed in (A) all neurons, or (B) motoneurons only, using the ELAV-GAL4 and OK6-GAL4 drivers respectively. Syncrip expression is robustly reduced when RNA interference is driven in all neurons, but non-significant when driven in motoneurons. Note that the ~38 kDa Syncrip band is almost entirely absent from central nervous tissue. Quantification performed on the ~50 kDa Syncrip band. Independent two-tailed Student's t-test; *** p<0.001 ** p<0.005 * p<0.05, n.s. p>0.05.

Biology Open (2014) 000, 1–11 doi:10.1242/bio.20149027

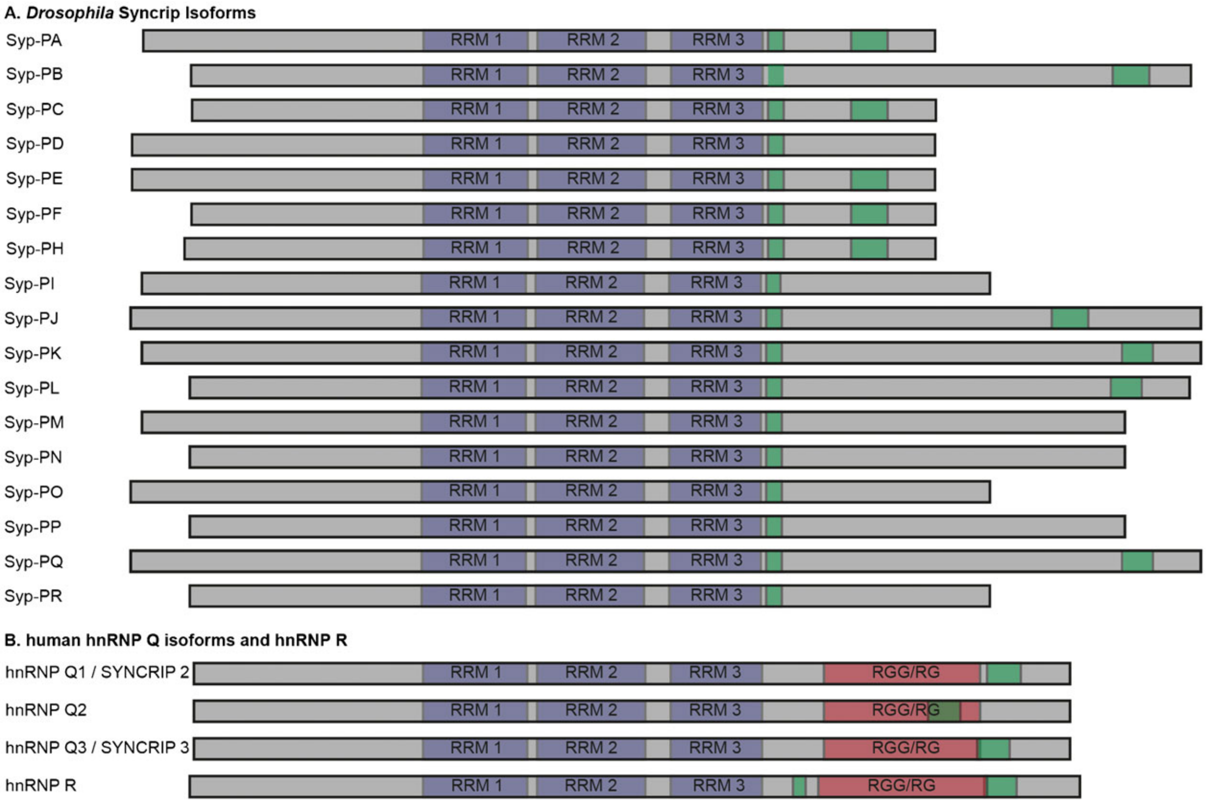

C. Syncrip homologues (% identity/similarity)

	Syp-PF	RRM1	RRM2	RRM3	RRM1-PC
hnRNP Q1 / SYNCRIP 2	47.1/59.6	61.5/74.4	66.2/81.8	56.9/80.0	67.6/81.1
hnRNP Q2	42.9/55.1	61.5/74.4	54.5/63.3	56.9/80.0	67.6/81.1
hnRNP Q3 / SYNCRIP 1	45.9/57.1	60.8/75.9	62.2/76.8	52.9/74.3	63.3/75.9
hnRNP R	46.0/58.2	64.9/81.1	64.9/83.1	56.9/78.5	64.9/79.7

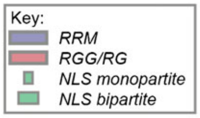

Fig. S6. *Drosophila* Syncrip is highly homologous to mammalian SYNCRIP, but lacks the RGG/RG domain. (A) There are 17 isoforms of *Drosophila* Syncrip (B) with similar domain organisation to mammalian SYNCRIP and hnRNPQ/R isoforms. *Drosophila* Syncrip however lacks the C-terminal domain containing RGG/RG motifs which is required for association with Synaptotagmins. (C) Despite this difference, the Syncrip and hnRNPQ/R RRM domains show high similarity and identity across *Drosophila* and mammalian isoforms.
